# A Case of Puerperal Appolectic Convulsions, with Spontaneous Expulsion of the Fœtus after Death

**Published:** 1858-10

**Authors:** Benjamin F. Fessenden

**Affiliations:** Plymouth, N. C.


*A Case of Puerperal Apoplectic Convulsions, with Spontaneous Expulsion of the Fœtus after Death.* By Benjamin F. Fessenden, M. D., Plymouth, N. C.

*June* 2, 1848.—I was called to Mrs. H——, a woman of medium stature, rather robust, aged thirty-one years. This was her third pregnancy; in the two first labors she did well, they were natural and terminated within the usual period. She had been in good health, but complained for the last month of headache, or a *bad feeling* about that organ. The day before I saw her, she complained of severe headache, and had a spell of blindness of short duration. At 8 o'clock P. M., she had symptoms of labor, being about the completion of her term. Her husband informed me that she was seized with violent convulsion about 10 o'clock, and that they had continued, with but slight intermissions, up to the moment of my arrival.

Owing to the distance from me, I did not see the patient until about 5 o'clock A. M., June 3, I then found her in a supine position, continually convulsed, every feature and gesture distorted, respiration attended with a hissing sound, frothing at the mouth and with each paroxysm lasting about ten minutes, after which she sank into a stertorous sleep. She was insensible, exhibiting no signs of consciousness or feeling; her pulse was full, hard and slow; the pupils of her eyes were dilated and insensible to light; her bowels were confined, and there was no uterine action during the time I was with her—if there had been any it was entirely suspended.

*Treatment of the Case.*—I immediately bled her, allowing the blood to flow until it made decided impression on the pulse. Gave her twelve grains of calomel, to be followed by a wineglassful of black draft every hour, until it operated on the bowels; and also recommended enemeta of infusion of senna, sulphate of magnesia and ol. terebinth. twelve ounces at a time, to be repeated until the bowels were thoroughly moved; with cold applications to the head. At 10 A. M., I sent for Dr. W. W. Ward in consultation. When he arrived about 2 o'clock P. M. there was no change in the condition of the patient; there had been no action from her bowels, and the enemas (three in number) were all retained. Upon consultation we applied cups to the temples and back of neck, blisters to back of neck and thighs, and gave two drops of ol. tiglii. On examination we found the os uteri still undilated and hard, and with no perceptible progress of the labor. She died about 6 o'clock P. M. The corpse was dressed as usual for the grave; and the day after, when about to put her in the coffiin, the child was found delivered. At what time this spontaneous action of the womb took place, I had no accurate means of knowing. On inquiry, I learned from those who were sitting up with the corpse, that about six or eight hours after it was dressed, there was an agitation or movement of the body, accompanied by a strange sound, which somewhat startled them at the time, but that attributing it to their imaginations, they thought no more of the matter until the discovery of the child. I suppose the expulsion must have taken place at that time.

[*North Carolina Medical Journal.*]

